# Unconventional band inversion and intrinsic quantum spin Hall effect in functionalized group-V binary films

**DOI:** 10.1038/s41598-017-05420-y

**Published:** 2017-07-21

**Authors:** Sheng-shi Li, Wei-xiao Ji, Ping Li, Shu-jun Hu, Tie Zhou, Chang-wen Zhang, Shi-shen Yan

**Affiliations:** 1 School of Physics, State Key Laboratory of Crystal Materials, Shandong University, Jinan, Shandong 250100 P.R. China; 2 School of Physics and Technology, University of Jinan, Jinan, Shandong 250022 P.R. China

## Abstract

Adequately understanding band inversion mechanism, one of the significant representations of topological phase, has substantial implications for design and regulation of topological insulators (TIs). Here, by identifying an unconventional band inversion, we propose an intrinsic quantum spin Hall (QSH) effect in iodinated group-V binary (ABI_2_) monolayers with a bulk gap as large as 0.409 eV, guaranteeing its viable application at room temperature. The nontrivial topological characters, which can be established by explicit demonstration of Z_2_ invariant and gapless helical edge states, are derived from the band inversion of antibonding states of *p*_x,y_ orbitals at the K point. Furthermore, the topological properties are tunable under strain engineering and external electric field, which supplies a route to manipulate the spin/charge conductance of edge states. These findings not only provide a new platform to better understand the underlying origin of QSH effect in functionalized group-V films, but also are highly desirable to design large-gap QSH insulators for practical applications in spintronics.

## Introduction

Topological insulators (TIs)^1–3^, a new quantum state of matter, which can effectively achieve the objective of spintronics by controlling spin configuration and spin current^4, 5^, have attracted great research enthusiasm since they were proposed in 2005. A fascinating hallmark of TIs is the existence of time-reversal-protected edge states or surface states which span insulating bulk state and show a robustness against nonmagnetic perturbations. In view of these advantages, TIs are quite promising for application in spintronics^6^ and quantum computations^7, 8^. To date, many three-dimensional (3D) materials have confirmed experimentally to be TIs, such as Bi_2_Se_3_^9^. However, tuning the Fermi level of 3D TIs still faces challenge, and the bulk carriers commonly exceed the surface carriers. By contrast, two-dimensional (2D) TIs, also known as quantum spin Hall (QSH) insulators, are more advantageous over 3D TIs since the electrons can only propagate along two directions with opposite spin and thus backscattering caused by nonmagnetic defects is completely forbidden, while the surface states of 3D TI are only free from exact 180° backscattering. Besides, from the perspective of practical application, 2D TIs can easily actualize functionality and integration of devices due to their lower dimensionality. Nevertheless, the experimental process with regard to 2D TIs confines to HgTe/CdTe^7, 8^ and InAs/GaSb^10^ quantum wells. The QSH effect of these quantum wells can only be observed under a harsh condition because of their small bulk gap, which severely blocks their application at room temperature. Hence, searching for novel 2D TIs which possess experimental feasibility and admirable QSH effect with large bulk gap is desirable.

Following the first proposed QSH insulator of graphene^11^, other 2D group-IV films, such as silicene^12^, germanene^12^, stanene^13, 14^ and plumbene^15^, are verified as 2D TIs successively. These hexagonal honeycomb structures provide a certain reference on searching for QSH insulator. For example, the graphene-like structures comprised of group-V element trigger considerable research attention, including Bi(111) bilayer (bismuthene)^16^, antimonene^17^ and arsenene^18^ films. Among them, the bismuthene is predicted as an intrinsic QSH insulator due to its stronger spin-orbit coupling (SOC) strength, and it has been successfully prepared on Bi_2_Te_3_ and Bi(111) substrates^16, 19, 20^. For atimonene and arsenene, which are normal insulators (NIs) in ground state, they can transform into 2D TIs with assistance of appropriate strain engineering^21–23^, accompanied with band inversion^24^ of *p*_x,y_-*p*_z_ at Γ point. Interestingly, similar results are also demonstrated in group-V binary monolayers, but they are thermodynamically instable with softened modes and imaginary frequency^25^. In fact, there also has possibility to prepare group-V binary monolayers, such as SbAs^26^, whose bulk material is naturally existing.

In experiments, the realization of strain engineering still faces challenges, especially for the operating range exceeding 10%. Chemical functionalization, as an alternative and useful route to introduce intrinsic topological phase, is proposed^14, 15, 27–34^. Such manipulation not only facilitates the regulation of electronic and topological properties, but also protects the desirable properties from environmental degradation. Recently, intensive efforts have implemented on functionalized group-V films^31, 33, 35–40^ which can readily achieve intrinsic nontrivial topology and shows classic Kane-Mele-type QSH effect. However, the topological mechanism of these systems is still a mystery topic to be further explored and understood. In view of above-mentioned group-V binary monolayers, two questions spontaneously arise: (i) can chemical functionalization simultaneously improve the stability and realize intrinsic QSH effect in group-V binary monolayers? (ii) If so, what is the potential topological mechanism, namely, how to characterize this topological nature?

In this work, based on first-principles calculations, we propose the simultaneous presence of unconventional band inversion and intrinsic QSH effect in iodinated group-V binary monolayers (ABI_2_) which are dynamically and thermally stable at room temperature. The topological phase, accompanied with a sufficiently large bulk gap of 0.409 eV, can be identified by the band inversion of antibonding states of *p*_x,y_ orbitals between A and B atoms induced by SOC at K point and explicit confirmation of Z_2_ invariant, as well as gapless helical edge states. In addition, their QSH effect can be modulated by strain engineering and external electric field with tunable bulk gap. The electronically controlled transition between nontrivial and trivial phases in AsBiI_2_ monolayer can effectively switch spin and charge transports to design topological quantum devices. These findings are conducive to propelling the understanding of topological mechanism in group-V films and supporting promising candidates to design spintronic and optoelectronic devices.

## Computational Details

All first-principles calculations based on density functional theory (DFT) are carried out using Vienna Ab-Initio Simulation Package (VASP)^41, 42^. The projector-augmented wave (PAW) method^43^ is used to describe the electron-ion potential, and the Generalized gradient approximation (GGA) in Perdew-Burke-Ernzerhof (PBE)^44, 45^ form is adapted to approximate the electron-electron interaction. The SOC interaction is included in the step of self-consistent calculations of electronic structure. The kinetic energy cutoff of plane wave basis is set as 500 eV. A unit cell with periodic boundary condition is employed, and a vacuum space of 20 Å is applied. We employ the k-meshes of 11 × 11 × 1 and 15 × 15 × 1 for geometry optimization and self-consistent electronic structure calculations, respectively. All lattice constants and atom coordinates are optimized with the self-consistent criteria of 10^−6^ eV until the convergence of force on each atom is less than 0.001 eV/Å. Hybrid HSE06 functional^46^ is used to confirm the band structure of functionalized systems. The phonon spectra are calculated using a supercell approach within the PHONOPY code^47^.

The calculations of edge states for ABI_2_ monolayers are implemented by Wannier 90 package^48^. Based on maximally localized Wannier functions (MLWFs), an iterative Green’s function method^49, 50^ is employed to calculate the edge states of a semi-infinite lattice and utilized to observer the local density of state (LDOS) of the edges.

## Results

**Figure 1:**
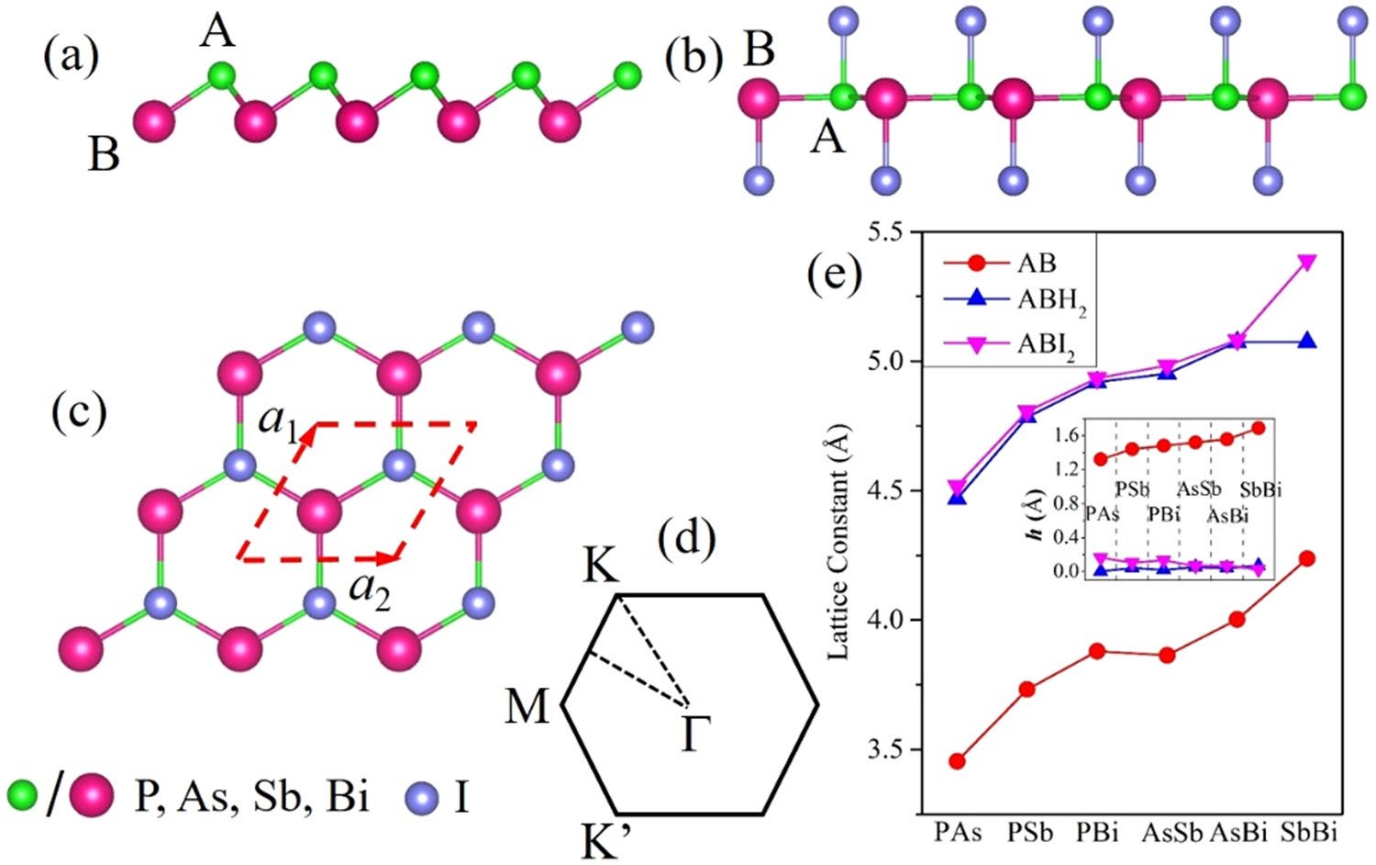
(**a**) Atomic structure of pristine AB monolayers. (**b**) Side view and (**c**) top view of ABI_2_ monolayer. (**d**) The corresponding 2D Brillouin zone of ABI_2_ monolayer with high-symmetry points. (**e**) Evolution of lattice constant for pristine AB, ABH_2_, ABI_2_ monolayers, respectively. The insert stands for the buckled height (*h*) as a function of different configurations.

The group-V binary monolayers, comprised of two elements from P to Bi, contain a total of six situations (PAs, PSb, PBi, AsSb, AsBi, SbBi). Here, they are collectively represented as AB monolayers, where A and B atoms are defined as the elements with minor and greater atomic number, respectively. Figure 1(a) depicts the side view of pristine AB monolayers, which obtains a buckled configuration. The AB monolayers are reported kinetically stable except that AsBi and SbBi monolayers possess softened modes and imaginary frequency near Γ point^25^. According to previously theoretical works^29, 51^, the QSH effect is strongly associated with the SOC strength of functional atoms, thus we choose to passivate the monolayers using iodine atom to maximize the SOC interaction. Here, the iodinated monolayers are denoted as ABI_2_ (PAsI_2_, PSbI_2_, PBiI_2_, AsSbI_2_, AsBiI_2_, SbBiI_2_), and their geometric structures are presented in Fig. 1(b,c), along with corresponding 2D Brillouin zone in Fig. 1(d). Compared with pristine cases, the ABI_2_ monolayers prefer a quasi-planar configuration with iodine atoms alternately decorated on both sides of AB monolayers (space group P3M1, No. 156). Actually, the case of iodination on one side is also investigated, the fact demonstrates the functionalized monolayers are unstable after structural optimization, which may be attributed to the coulomb repulsion between iodine atoms and its smaller electronegativity. So we primarily concentrate on the configuration with decoration on both sides. In Fig. 1(e), we present the lattice constants of pristine and functionalized AB monolayers. The former is in good agreement with previous results^25^, while the latter indicates that AB monolayers are stretched under the effect of iodination, accompanied with buckled height (*h*) reducing to around 0.1 Å, as illustrated in the insert of Fig. 1(e). From Table 1, one can also see that, as the rise of atomic number of component atoms, both lattice constant and A-B bond length increase gradually in order of PAsI_2_ < PSbI_2_ < PBiI_2_ < AsSbI_2_ < AsBiI_2_ < SbBiI_2_.

**Table 1:** Calculated lattice constant (*a*), bond length (*d*_A−B_), buckled height (*h*), formation energy (*E*_*f*_) and material property of ABI_2_ monolayers.

Configurations	*a* (Å)	*d*_A−B_ (Å)	*h* (Å)	*E*_*f*_ (eV/unit cell)	*E*_k_ (eV)	*E*_g_ (eV)	${{\boldsymbol{E}}}_{{\boldsymbol{g}}}^{{\boldsymbol{SOC}}}$ (eV)	Property
PAsI_2_	4.517	2.613	0.161	−1.901	0.443	0.443	0.147	NI
PSbI_2_	4.807	2.777	0.101	−2.452	0.828	0.788	0.567	NI
PBiI_2_	4.935	2.853	0.133	−2.437	1.258	1.225	0.320	NI
AsSbI_2_	4.983	2.877	0.064	−3.287	0.402	0.402	0.082	NI
AsBiI_2_	5.082	2.935	0.067	−3.267	0.831	0.831	0.067	TI
SbBiI_2_	5.389	3.112	0.024	−3.669	0.380	0.380	0.409	TI

*E*_k_ and *E*_g_ represent the band gap opening at K point and global band gap excluding SOC, respectively. ${E}_{g}^{SOC}$ denotes the SOC-induced band gap.

**Figure 2:**
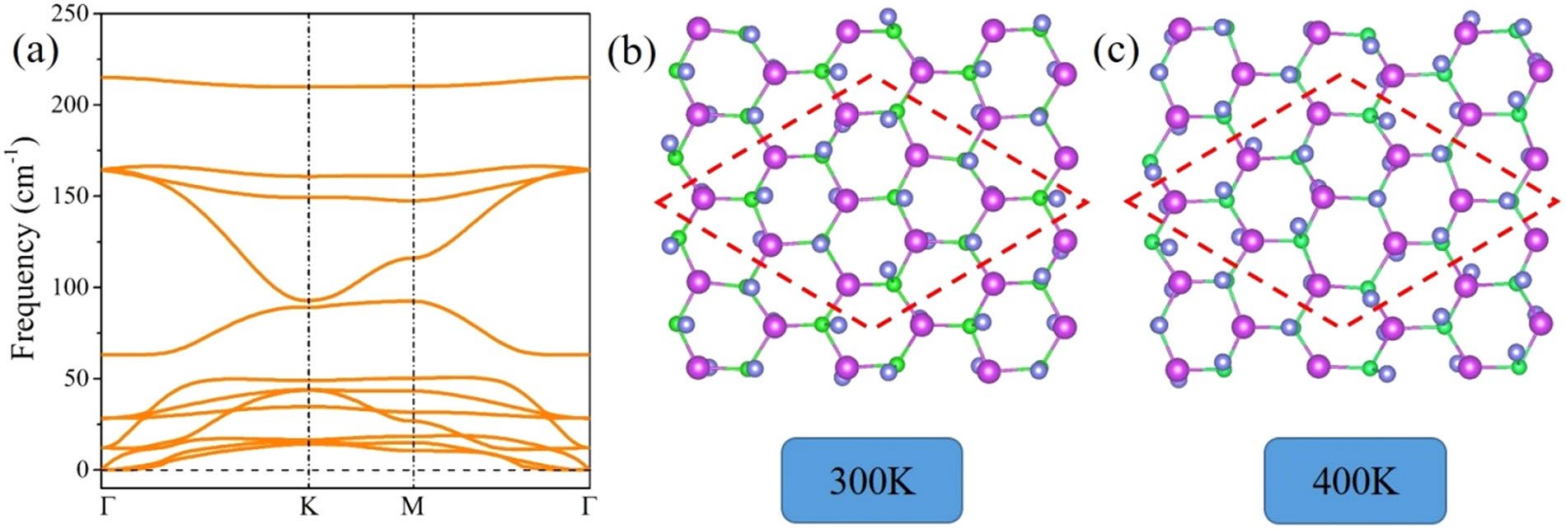
(**a**) Calculated phonon spectrum of AsBiI_2_ monolayer. Corresponding molecular dynamics (MD) simulation for a 3 × 3 supercell at 300 K (**b**) and 400 K (**c**) after 3 ps, respectively.

The thermodynamic stability of ABI_2_ monolayers is evaluated by the formation energy which is defined as:

1 $${E}_{f}=E({{\rm{ABI}}}_{2})-{n}_{{\rm{A}}}{E}_{{\rm{A}}}-{n}_{{\rm{B}}}{E}_{{\rm{B}}}-{n}_{{\rm{I}}}{E}_{{\rm{I}}},$$

where *E*(ABI_2_) represents the total energies of iodinated AB monolayers. *E*_A_, *E*_B_ and *E*_I_ are the chemical potential of A, B and iodine atoms obtained from bulk bismuth, bulk antimony, bulk arsenic, black phosphorus and bulk iodine, respectively. The *n*_A_, *n*_B_ and *n*_I_ are the numbers of A, B and iodine atoms in the ABI_2_ unit cell. The calculated formation energies are listed in Table 1. The negative values demonstrate that the iodination is an exothermal process, suggesting a higher thermodynamic stability. Moreover, the calculation of phonon spectrum is performed to check the kinetic stability of ABI_2_ monolayers, and we find that the iodination can effectively remove the softened modes and imaginary frequency in AsBi and SbBi monolayers, as shown in Fig. 2(a) and Fig. S1 in Supplementary Information. Similar results are also established for the other configurations, as depicted in Fig. S1.

On the other hand, we perform *ab initio* molecular dynamics (MD) simulations using a 3 × 3 × 1 supercell at various temperature with a time step of 2 fs to explore the structural perturbation of ABI_2_ monolayers during the 3 ps simulation. Taking AsBiI_2_ monolayer as example, the honeycomb lattice deforms slightly at 300 K in Fig. 2(b). As the temperature increases to 400 K, the geometric structure suffers a serious distortion, but no bond breaking can be observed, as illustrated in Fig. 2(c), indicating that the AsBiI_2_ monolayer could be steadily survived at room temperature. The thermal stabilities for other ABI_2_ systems are also displayed in Fig. S1. As a consequence, all ABI_2_ monolayers possess favorable stability, which is very significant for practical applications.

To reveal the prominent influence of iodination on the electronic properties of AB monolayers, we firstly present the calculated band structures of pristine AB monolayers in Fig. S2. Regardless of SOC, they exhibit semiconducting properties, while the introduction of SOC just modifies the size of band gap due to band splitting. As a result, the QSH effect is absent for AB monolayers, indicating a trivial topological phase. Through projecting the energy bands with atomic orbitals, one can see that the states near the Fermi level at the Γ point mainly derive from the *p*_z_ and *p*_x,y_ orbitals.

**Figure 3:**
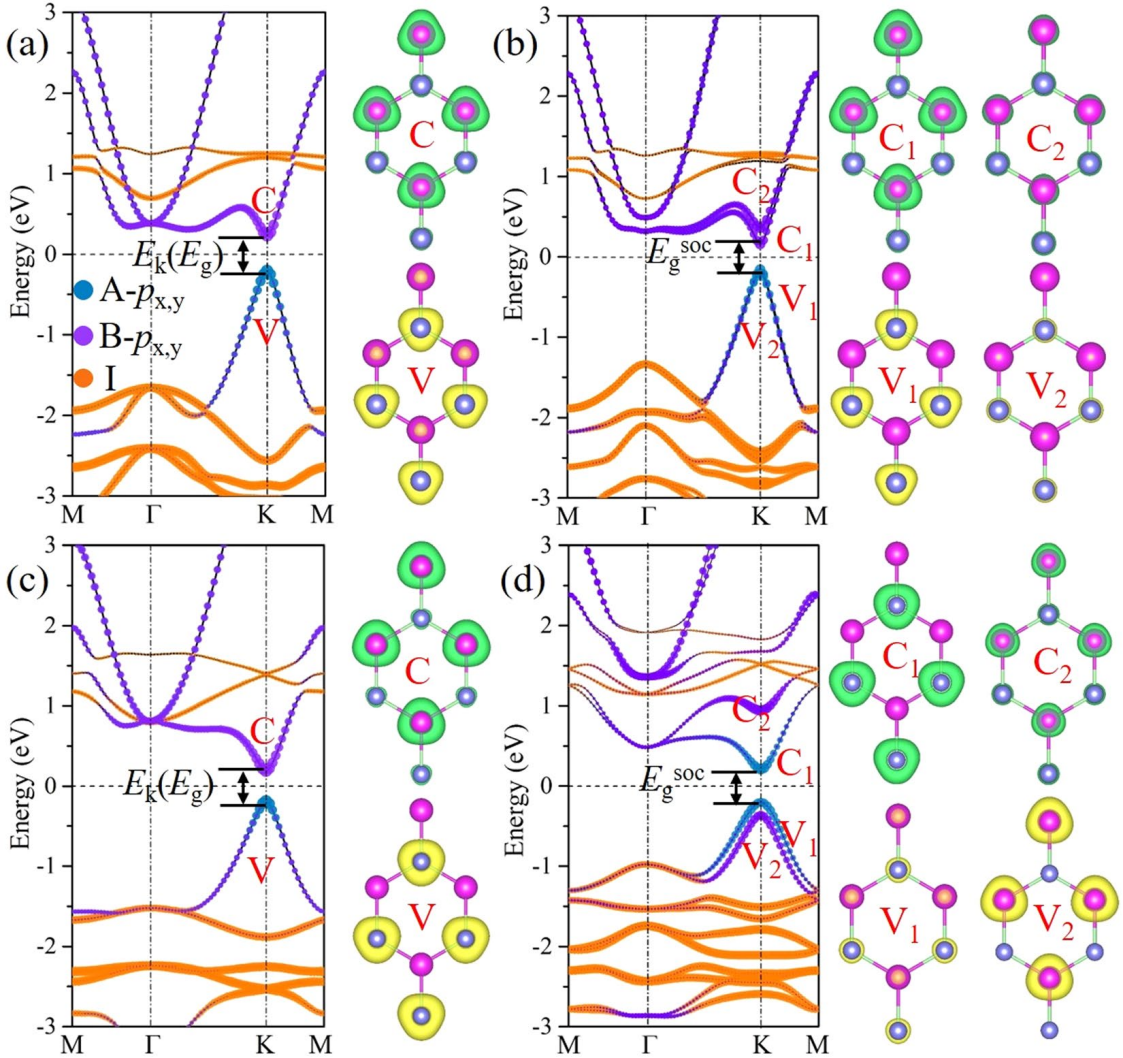
Orbital-resolved band structures and partial charge density of VBM and CBM. (**a**) PAsI_2_ monolayer without SOC, (**b**) PAsI_2_ monolayer with SOC, (**c**) SbBiI_2_ monolayer without SOC and (**d**) SbBiI_2_ monolayers with SOC.

Next, we turn to the electronic properties of ABI_2_ monolayers. Here, the PAsI_2_ and SbBiI_2_ are chosen as the representatives and their band structures are presented in Fig. 3. For PAsI_2_ monolayer, it shows a semiconducting property with a direct band gap (*E*_K_) of 0.443 eV located at the K point when excluding SOC, which is distinctly different from the semi-metallicity in functionalized Bi(111) bilayer^31, 38–40, 52^, as illustrated in Fig. 3(a). Similar band features can be obtained in other configurations, except for PSbI_2_ and PBiI_2_ monolayers which show indirect global band gaps (*E*_g_) due to the deviation of conduction bands minimum (CBM), see Fig. S3. As the SOC is taken into account, the most conspicuous change is the removal of degeneracy of energy bands owing to the lack of spatial inversion symmetry. We find that the valence bands maximum (VBM) and CBM of ABI_2_ monolayers are all fixed at K point, forming a direct band gap (${E}_{g}^{SOC}$). Compared with the cases without SOC, the band gaps of ABI_2_ monolayers are decreased except for SbBiI_2_ monolayer, as listed in Table 1.

In order to get a deeper insight into the band structure feature, we project energy bands onto different atomic orbitals. Regardless of SOC, for PAsI_2_ monolayer, the P-*p*_x,y_ and As-*p*_x,y_ orbitals are distinctly separated in the vicinity of Fermi level and contribute to the VBM and CBM, respectively. The partial charge densities with respect to VBM and CBM are further calculated. One can see that the VBM and CBM are localized at the P and As sites, respectively, as shown in Fig. 3(a). When considering SOC effect, the contributions of atomic orbitals at VBM and CBM are not altered, as evidenced in Fig. 3(b). Similar to PAsI_2_ monolayer, the VBM and CBM of SbBiI_2_ monolayer are derived from Sb-*p*_x,y_ and Bi-*p*_x,y_ orbitals without SOC, as shown in Fig. 3(c). However, the stronger SOC effect triggers the realignment of band order. Here, both VBM and CBM are mainly originated from the Sb-*p*_x,y_ orbitals, whereas the energy band contributed by Bi-*p*_x,y_ orbitals reverses into valance band, being the second highest occupied state, see Fig. 3(d). The partial charge densities of these four bands at K point are in good agreement with the orbital-resolved band structure. Such SOC induced band inversion between Sb-*p*_x,y_ and Bi-*p*_x,y_ strongly points to the presence of QSH effect. Similar results can be observed in AsBiI_2_ monolayer, while the other monolayers are not provided with this form of band inversion, as shown in Fig. S3.

**Figure 4:**
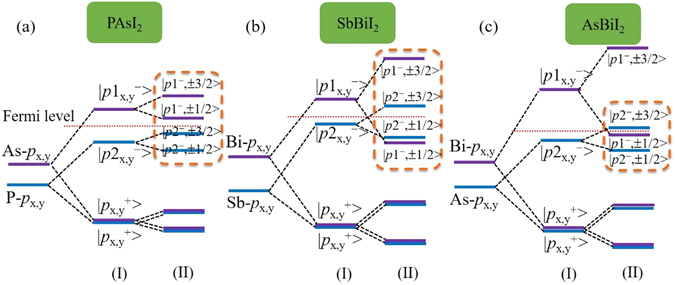
The evolution of *p*_x,y_ orbitals of A and B atoms at K point near the Fermi level under chemical bonding (I) and SOC (II) effects. (**a**) PAsI_2_, (**b**) SbBiI_2_, and (**c**) AsBiI_2_ monolayers.

To understand the physical mechanism underlying the band inversion, we turn to the effects of chemical bonding and SOC on energy bands at K point for ABI_2_ monolayers and the schematic diagrams are illustrated in Fig. 4. Here, only the evolution of *p*_x,y_ orbitals of A and B atoms is focused on near the Fermi level, since the *p*_z_ orbitals are saturated by iodine atoms. Starting from PAsI_2_ monolayer, in stage (I), the formation of chemical bonding makes the *p*_x,y_ orbitals split into bonding and antibonding states, i. e., $|{p}_{{\rm{x}},{\rm{y}}}^{+}\rangle$ and $|{p}_{{\rm{x}},{\rm{y}}}^{-}\rangle$, where the superscripts + and − represent the bonding and antibonding states. For convenience, the states near the Fermi level are labeled as $|p{1}_{{\rm{x}},{\rm{y}}}^{-}\rangle$ and $|p{2}_{{\rm{x}},{\rm{y}}}^{-}\rangle$, as illustrated in Fig. 4(a), which are respectively originated from As and P atoms. The $|p{1}_{{\rm{x}},{\rm{y}}}^{-}\rangle$ locates above $|p{2}_{{\rm{x}},{\rm{y}}}^{-}\rangle$, yielding a sizeable band gap. When SOC is turned on in stage (II), the degeneracy of the $|{p}_{{\rm{x}},{\rm{y}}}^{-}\rangle$ is lifted, resulting in the decrease of the band gap, but the sequence of energy level is unchanged, as shown in Fig. 4(a). Similar situation is obtained in PSbI_2_, PBiI_2_ and AsSbI_2_ monolayers. For SbBiI_2_ monolayer, a greater orbital splitting is generated due to its larger strength of SOC^53^, driving |*p*1^−^, ±1/2〉 directly downshift below |*p*2^−^, ±1/2〉 and improving |*p*2^−^, ±3/2〉 above the Fermi level, thus introduces a significant band inversion, see Fig. 4(b). Meanwhile, we find that the AsBiI_2_ monolayer also possesses band inversion, in which the |*p*1^−^, ±1/2〉 and |*p*2^−^, ±3/2〉 are obviously reversed at the Fermi level, as shown in Fig. 4(c). In a word, the appearance of band inversion, to a great extent, is determined by the bonding and SOC strength of A and B atoms. It is important to notice that such band inversion associated with antibonding states of *p*_x,y_ orbitals for AsBiI_2_ and SbBiI_2_ monolayers is distinctly different from the cases of functionalized III-V^30^ and IV films^14, 15, 24, 34, 54^, indicating that it is an unconventional mechanism to predict 2D TIs.

A QSH insulator is usually represented by a topological invariant Z_2_ = 1. Thus, to verify our supposition, we calculate the Z_2_ invariant of ABI_2_ monolayers by the scheme proposed by Soluyanov and Vanderbilt^55, 56^. This method tracks the evolution of Wannier Center of Charges (WCCs) for an effective 1D system with fixed *k*_y_ in the subspace of occupied bands. The Wannier functions (WF) in regard to lattice vector *R* can be written as:

2 $$|R,n\rangle =\frac{1}{2\pi }{\int }_{-\pi }^{\pi }dk{e}^{-ik(R-x)}|{u}_{nk}\rangle .$$

The WF depends on a gauge choice for the Bloch states |*u*_*nk*_〉. A WCC ${\bar{x}}_{n}$ is defined as the mean value of $\langle 0n|\hat{X}|0n\rangle$, where the $\hat{X}$ is the position operator and |0*n*〉 is the state corresponding to a WF in the cell with *R* = 0. Then we can obtain that:

3 $${\bar{x}}_{n}=\frac{i}{2\pi }{\int }_{-\pi }^{\pi }dk\langle {u}_{nk}|{\partial }_{k}|{u}_{nk}\rangle .$$

**Figure 5:**
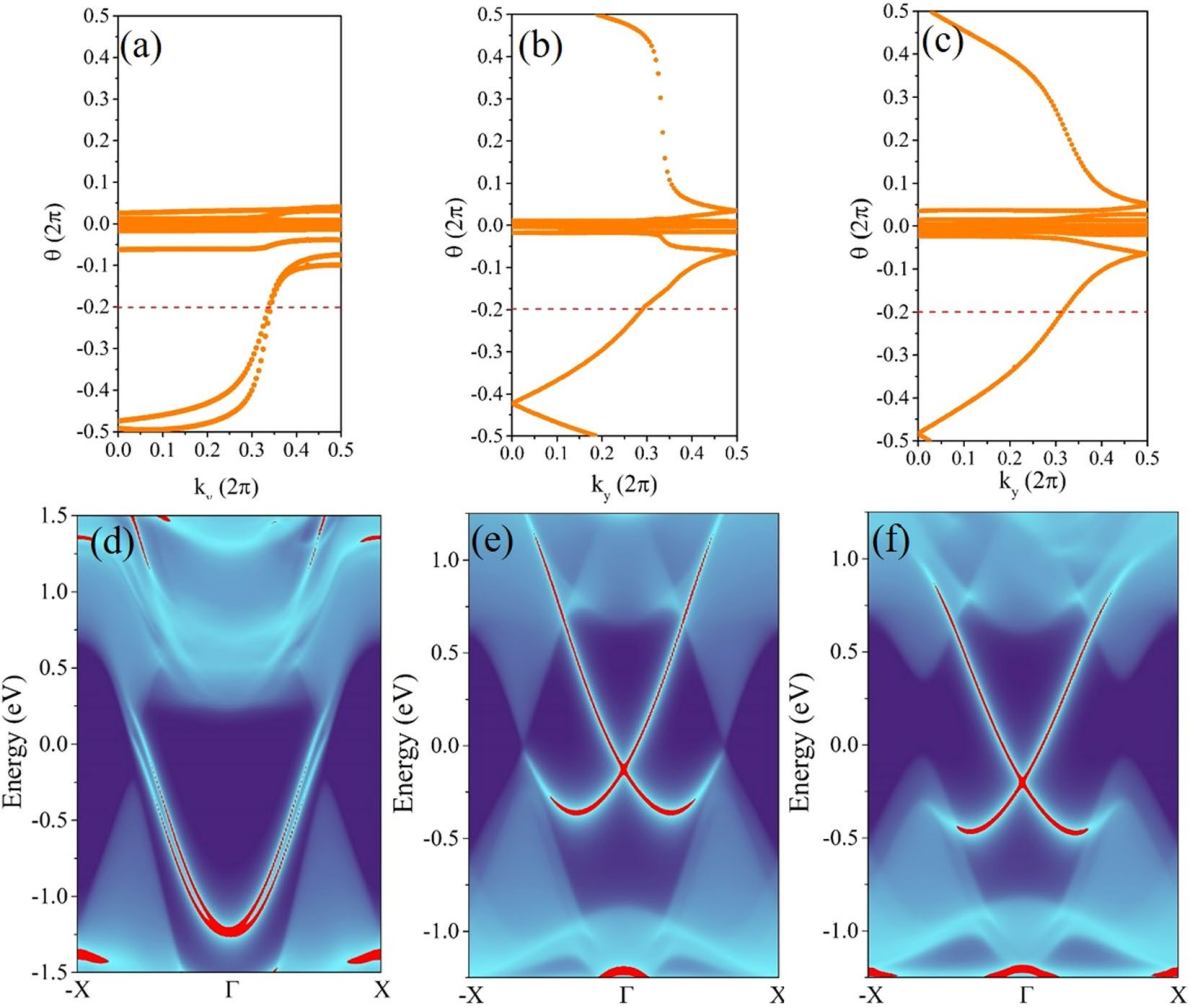
Evolution of the Wannier charge centers (WCCs) along *k*_y_ for (**a**) PAsI_2_, (**b**) AsBiI_2_ and (**c**) SbBiI_2_ monolayer. Calculated semi-infinite edge states of (**d**) PAsI_2_, (**e**) AsBiI_2_ and (**f**) SbBiI_2_ monolayers.

Assuming that ${\sum }_{\alpha }{\bar{x}}_{\alpha }^{S}=\frac{1}{2\pi }{\int }_{BZ}{A}^{S}$ with *S* = *I* or *II*, where α is a band index of the occupied states and *I* and *II* are the Kramer partners, while *A* is the Berry connection. So the Z_2_ topological invariant can be expressed as:

4 $${Z}_{2}=\sum _{\alpha }[{\bar{x}}_{\alpha }^{I}(TRI{M}_{1})-{\bar{x}}_{\alpha }^{II}(TRI{M}_{1})]-\sum _{\alpha }[{\bar{x}}_{\alpha }^{I}(TRI{M}_{2})-{\bar{x}}_{\alpha }^{II}(TRI{M}_{2})],$$

where TRIM represents the time-reversal-invariant momentum. The identification of Z_2_ invariant can be obtained by counting the numbers of crossing between any arbitrary horizontal reference line and evolution of the WCCs, where the odd and even numbers represent nontrivial and trivial topological phase, respectively. Figure 5(a–c) present the evolution of the WCCs between two TRIM of the Brillouin zone for PAsI_2_, AsBiI_2_, and SbBiI_2_ monolayers. Corresponding to above analysis, both AsBiI_2_ and SbBiI_2_ monolayers are nontrivial QSH insulators with Z_2_ = 1. However, the PAsI_2_ monolayer and the other configurations obtain a Z_2_ invariant of 0, as depicted in Fig. S4, indicating a trivial topological phase.

The presence of topologically protected and spin-momentum locked edge states is one of the prominent features of QSH insulators. The LDOSs of edges for ABI_2_ monolayers are further calculated to confirm the topological properties exactly. For PAsI_2_ monolayer, as shown in Fig. 5(d), the edge states start from conduction band and then are absorbed back into the same conduction band rather than providing spin transport channels between valence and conduction bands, evidencing that PAsI_2_ monolayer is a trivial insulator. Similar LDOSs are verified in PSbI_2_, PBiI_2_, and AsSbI_2_ monolayers, see Fig. S5(a–c), indicating that they likewise belong to the scope of NIs. On the contrary, we notice that a pair of gapless edge states traverse across the bulk gap and connect the conduction and valence bands in AsBiI_2_ and SbBiI_2_ monolayers, as depicted in Fig. 5(e,f). By projecting the contribution of spin up and spin down component, see Fig. S5(d,e), we find that the spin-momenta of those states are locked, suggesting the counter-propagating edge states have opposite spin-polarization. Thus, we can readily ascertain the nontrivial topological phase in AsBiI_2_ and SbBiI_2_ monolayers. Moreover, apart from intrinsic QSH effect, the satisfactory bulk gaps guarantee their experimental observability and viable application at room temperature, especially for SbBiI_2_ monolayer, whose bulk gap attains a sufficiently large value of 0.409 eV.

**Figure 6:**
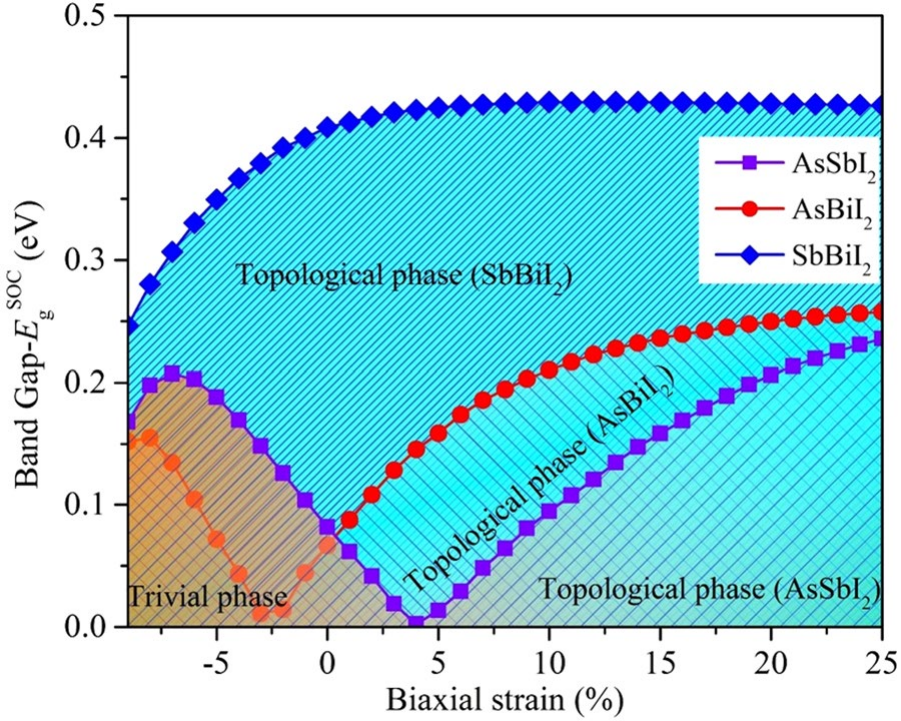
Variation of band gap (${E}_{g}^{SOC}$) as a function of biaxial strain for AsSbI_2_, AsBiI_2_, and SbBiI_2_ monolayers, respectively.

According to former analysis, under a fixed SOC strength, the band inversion also relates to the bond strength which can be tuned by strain engineering. To realize the transition of TI and enhance the bulk gaps, we employ in-plane biaxial strain on ABI_2_ monolayers, which equals changing the lattices as *ε* = (*a* − *a*_0_)/*a*_0_, where *a* (*a*_0_) is lattice constant under the strain (equilibrium) condition. Figure 6 presents the variation of band gap as a function of biaxial strain. One can see that the SbBiI_2_ monolayer maintains its topological nature in the range of −9~20%. Under tensile strain, the band gap increases monotonously and reaches a maximum of 0.429 eV at 5%. After that, it almost remains unchanged even if the tensile strain reaches 25%. Instead, the compressive strain leads to the decrease of band gap. It can be interpreted by the fact that the shorter bond length drives the energy difference between $|p{2}_{{\rm{x}},{\rm{y}}}^{-}\rangle$ and $|p{1}_{{\rm{x}},{\rm{y}}}^{-}\rangle$ to reduce, and then the band inversion similar to AsBiI_2_ monolayer arises under SOC effect. To be brief, the SbBiI_2_ monolayer shows a favorable robustness of QSH effect against strain engineering. However, for AsBiI_2_ monolayer, the QSH effect can be easily tuned by strain engineering due to its relatively weak inversion strength. From Fig. 6, one can see that the band gap decreases firstly and then increases with the increasing compressive strain, in which the critical point of −3% indicates the annihilation of band inversion between |*p*2^−^, ±3/2〉 and |*p*1^−^, ±1/2〉, resulting in a trivial topological phase. On the contrary, the tensile strain enlarges the band gap significantly, persisting the topological nature. Interestingly, the AsSbI_2_ monolayer, a NI at equilibrium state, can transform into QSH insulator under tensile strain at 4%, and the continuously increasing strain would improves the band gap, demonstrating its potential application in spintronic devices. Furthermore, the tunability of the band gap and TI phase transition for PAsI_2_, PSbI_2_ and PBiI_2_ monolayers are also presented in Fig. S6. Owing to their rather weak SOC strength, the achievement of band inversion requires sufficiently small energy difference of $|{p}_{{\rm{x}},{\rm{y}}}^{-}\rangle$ between A and B atoms, which means that a prodigious tensile strain should be employed. The critical points for these configurations are 17%, 21% and 9%, respectively, which are conducive to understand the physical mechanism rather than application in devices, because such large tensile strain still faces many challenges in experiments.

**Figure 7:**
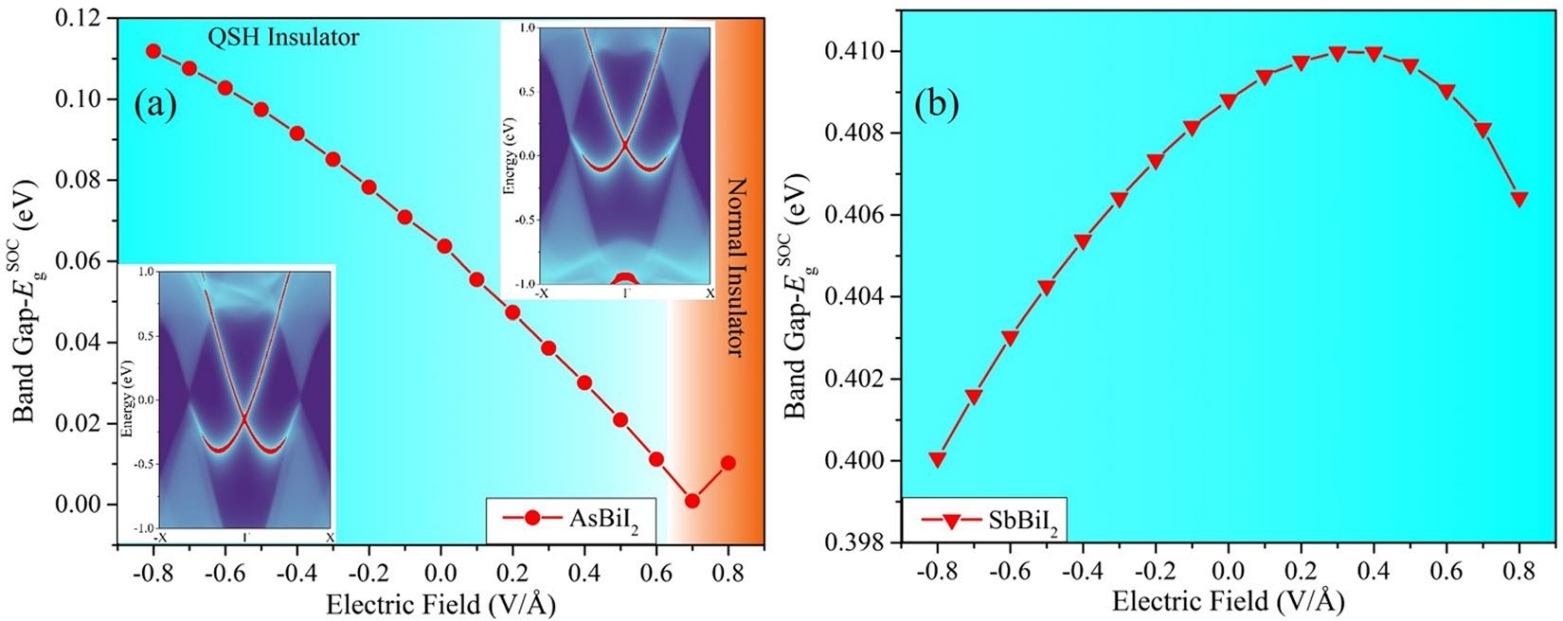
Variation of band gap (${E}_{g}^{SOC}$) as a function of external electric field for (**a**) AsBiI_2_ and (**b**) SbBiI_2_ monolayers, respectively. The left and right inserts of (**a**) are LDOSs of edges at −0.5 V/Å and 0.8 V/Å, respectively.

The facile mechanism of band inversion also provides a possibility to control the topological properties of ABI_2_ monolayers by external electric field. Taking AsBiI_2_ and SbBiI_2_ monolayers as representatives, Fig. 7(a,b) present the variation of band gaps with respect to an external electric field perpendicular to the films. For AsBiI_2_ monolayer [Fig. 7(a)], the intrinsic QSH effect is persevered under the negative electric field, in which the band gap increases monotonically. Under positive electric field, the band gap drops rapidly and reduces to zero at a critical field of 0.7 V/Å, while it would open up again for larger electric field, suggesting a trivial topological phase transition. It is feasible to achieve the control of on/off functions about spin and charge in conducting channels by external electric field, which has substantial implications for promoting the development of QSH devices^5, 57–61^, such as topological field-effect transistor (TFET). Here, we propose a conceptual design of TFET based on van der Waals heterostructures which are constructed by AsBiI_2_ monolayer and substrates with large band gap, such as *h*-BN, as shown in Fig. S7. In general, the van der Waals interaction has little influence on topological properties of monolayers, thus such multilayer structure can effectively increase the number of edge transport channels. The vertical electric field induced by the top and bottom gates can easily realize the on/off function. Additionally, we find that the topological phase of SbBiI_2_ monolayer is preserved under external electric field, manifesting an excellent robustness of QSH effect. The band gap shows a parabolic tendency with a maximum of 0.410 eV at 0.3 V/Å, which may be attributed to the competition between external electric field and internal polarized field. For PAsI_2_, PSbI_2_, PBiI_2_ and AsSbI_2_ monolayers, no topological transition can be observed under electric field, while their band gaps can be effectively tuned with a linear variation, as shown in Fig. S8. According to Bader analysis, we find the dipole moment of monolayers in vertical direction is along the positive orientation of z axis. When the negative electric field is applied, the dipole moment would be enhanced correspondingly due to the electron transfer from B to A atom, leading to the degeneracy further lifted. Thus, the energy levels near the Fermi level shift toward each other and the band gap decreases continuously. However, the positive electric field plays an opposite role. Such tunable band gap is highly favorable for applications in optoelectronic devices.

## Discussion

Having established the existence of QSH phase in iodinated group-V binary monolayers, to further characterize the unconventional mechanism of band inversion, we investigate the variation of band gap at K point for all ABI_2_ monolayers as a function of SOC strength λ/λ_0_, which is artificially set partial fractions of the original SOC strength λ_0_, as shown in Fig. S9. The band gaps of SbBiI_2_ and AsBiI_2_ monolayers close at the relative SOC strength of 0.4 and 0.9, respectively, and then reopen with larger SOC, indicating the occurrence of band inversion and topological phase. Similar result has been reported in Bi-III monolayers^62^, but it is distinctly different from the functionalized group-V films whose band gap would continuously increase with relative SOC strength. For AsSbI_2_ and PBiI_2_ monolayers, the band gaps reduce to zero when the λ/λ_0_ are 1.2 and 1.7, respectively, suggesting that these two monolayers also have the feasibility to transform into QSH insulators by doping isoelectronic heavier elements with stronger SOC strength. However, until the relative SOC strength reaching 2.0, the band gaps closing and reopening cannot be observed for PSbI_2_ and PAsI_2_ monolayers due to their inherently weak SOC strength.

As compared with pristine AB monolayers, the intrinsic QSH effect in ABI_2_ monolayers is induced by I decoration. In fact, the states contributed by iodine atoms are far away from the Fermi level, and the role of iodination is orbital filtering effect (OFE), which has been reported in many kinds of 2D films^14, 15, 27–32, 34, 38^. Considering that other halogen atoms (F, Cl, and Br) and hydrogen atom are also feasible to achieve these effects, we wonder that: is the QSH effect relevant to decorated atom? If so, can the QSH effect still be characterized by such unconventional band inversion?

To verify it, we further investigate the electronic properties of hydrogenated AB monolayers (ABH_2_). Their structural parameters are listed in Table S1 and illustrated in Fig. 1(e). One can see that the lattice constants and bond lengths of ABH_2_ monolayers are roughly equal to that in ABI_2_ monolayer, which also belong to space group P3M1. The optimized *h* of ABH_2_ monolayers, however, becomes smaller clearly, except for SbBiH_2_, implying that the A-B bond strength in ABH_2_ monolayers would be stronger than ABI_2_ monolayers. In the light of orbital-resolved band structures without SOC, as presented in Fig. S10, we can see that the orbital contributions in ABH_2_ monolayers are analogy to ABI_2_ monolayers, i.e., the states near the Fermi level are still determined by $|{p}_{{\rm{x}},{\rm{y}}}^{-}\rangle$ orbitals. Compared with ABI_2_ monolayers, with the exception of SbBi, the band gaps of ABH_2_ are smaller, see Table S1, which is mainly attributed to the energy-level shift induced by bond strength. After considering SOC effect, the band gaps of ABH_2_ monolayers decrease due to band splitting, as shown in Fig. S11. Under this circumstance, the above-mentioned band inversion between |*p*2^−^, ±3/2〉 and |*p*1^−^, ±1/2〉 is only applicable to the SbBiH_2_ monolayer, which strongly points to the presence of QSH effect. For AsBiH_2_ monolayer, though the energy difference between $|p{2}_{{\rm{x}},{\rm{y}}}^{-}\rangle$ and $|p{1}_{{\rm{x}},{\rm{y}}}^{-}\rangle$ orbitals is much smaller, it is still irrelevant to band inversion. As a consequence, the emergence of topological phase in AB monolayers is also dependent on the functional atoms. Besides, the other hydrogenated AB monolayers also exhibit a trivial band order. The LDOS of edges for these ABH_2_ monolayers are further calculated and presented in Fig. S12. Indeed, only SbBiH_2_ monolayer shows the gapless helical edge states, spanning the bulk gap, which evidences the nontrivial topological nature. In a word, the intrinsic QSH effect of these AB monolayers is sensitive to the functional atoms, especially for AsBi, and the unconventional band inversion can effectively characterize the nontrivial topological phase.

As is well known, the GGA exchange potential usually underestimates the band gap of semiconductors or insulators. To ensure the accuracy of band gap calculation, the more reliable hybrid HSE06 functional is adopted including the SOC effect. Here, the AsBiI_2_ and AsBiH_2_ monolayers are checked due to their smallest band gap in iodinated and hydrogenated systems, and the orbital projected band structures are presented in Fig. S13(a,b). We find that the band gap of AsBiI_2_ monolayer reduces to 0.019 eV. Combined with the atomic orbital contributions, this scenario can be understood by the fact that the band inversion vanishes with HSE06 correction, namely, a trivial topological phase formed. Even so, the nontrivial topology of AsBiI_2_ monolayer can be achieved easily with assistance of external factor, such as strain engineering, which still possesses the above excellent properties and potential application. However, for AsBiH_2_ monolayer, the band gap increases to 0.433 eV, maintaining its NI phase. Nevertheless, the calculated band structure of SbBiH_2_ with HSE06 functional is different from above cases, as shown in Fig. S13(c). Though its band gap is enhanced up to 0.545 eV, the band inversion between $|{p}_{{\rm{x}},{\rm{y}}}^{-}\rangle$ orbitals of Sb and Bi atoms is preserved, revealing that the band topology is robust.

## Conclusions

In summary, we perform first-principles calculations to demonstrate the intrinsic QSH effect in AsBiI_2_ and SbBiI_2_ monolayers, which can be characterized by a unconventional band inversion of $|{p}_{{\rm{x}},{\rm{y}}}^{-}\rangle$ orbitals induced by SOC at K point. This nontrivial topological phase is verified by the explicit confirmation of Z_2_ invariant and gapless helical edge states, with a sizable bulk gap of 0.409 eV, which is sufficiently large for practical application at room temperature. Their topological properties can be effectively tuned by external factors, such as strain engineering and electric field, in which the electronically dominated transition between nontrivial and trivial phase supplies an avenue for manipulating the spin/charge conductance of edge state. These findings are of fundamental importance to theoretical and experimental studies of group-V binary monolayers, which provides an ideal platform to design optoelectronic and QSH devices.

## Electronic supplementary material

Supplementary Information
