# Supplementary material for: Unconventional band inversion and intrinsic quantum spin Hall effect in functionalized group-V binary films
Source: Sci Rep. 2017 Jul 21;7:6126. doi: 10.1038/s41598-017-05420-y (PMC5522398; doi:10.1038/s41598-017-05420-y)
Supplement: Supplementary file 1 — Supplementary Information [file 41598_2017_5420_MOESM1_ESM.doc]

Supplementary Information for:

**Unconventional band inversion and intrinsic quantum spin Hall effect in functionalized group-V binary films**

Sheng-shi Li^1^, Wei-xiao Ji^2^, Ping Li^2^, Shu-jun Hu^1^, Tie Zhou^1^, Chang-wen Zhang^2^*, Shi-shen Yan^1^*

**Corresponding Authors:**

*E-mail: zhchwsd@163.com

*E-mail: [shishenyan@sdu.edu.cn](mailto:shishenyan@sdu.edu.cn)

This file includes:

Table S1

Figure S1 to S13

Table S1 Calculated lattice constant (*a*), bond length (*d*_A-B_), buckled height (*h*), formation energy (*E_f_*) and material property of ABH_2_ monolayers. *E*_k_ and *E*_g_ represent the band gap opening at the K point and global band gap excluding SOC, respectively. *E*_g_^SOC^ denotes the SOC-induced band gap.

| **Configu-**  **rations** | ***a***  **(Å)** | ***d*_A-B_**  **(Å)** | ***h***  **(Å)** | ***E_f_***  **(eV/unit cell)** | ***E*_k_**  **(eV)** | ***E*_g_**  **(eV)** | ***E*_g_^SOC^**  **(eV)** | **Property** |
| --- | --- | --- | --- | --- | --- | --- | --- | --- |

| **PAsH_2_** | 4.469 | 2.580 | 0.001 | -5.679 | 0.394 | 0.383 | 0.281 | NI |
| --- | --- | --- | --- | --- | --- | --- | --- | --- |
| **PSbH_2_** | 4.785 | 2.763 | 0.044 | -5.830 | 0.738 | 0.704 | 0.499 | NI |
| **PBiH_2_** | 4.920 | 2.841 | 0.019 | -5.573 | 1.184 | 0.704 | 0.438 | NI |
| **AsSbH_2_** | 4.952 | 2.859 | 0.055 | -6.376 | 0.337 | 0.337 | 0.032 | NI |
| **AsBiH_2_** | 5.074 | 2.930 | 0.038 | -6.124 | 0.783 | 0.783 | 0.016 | NI |
| **SbBiH_2_** | 5.398 | 3.117 | 0.070 | -6.336 | 0.434 | 0.434 | 0.384 | TI |


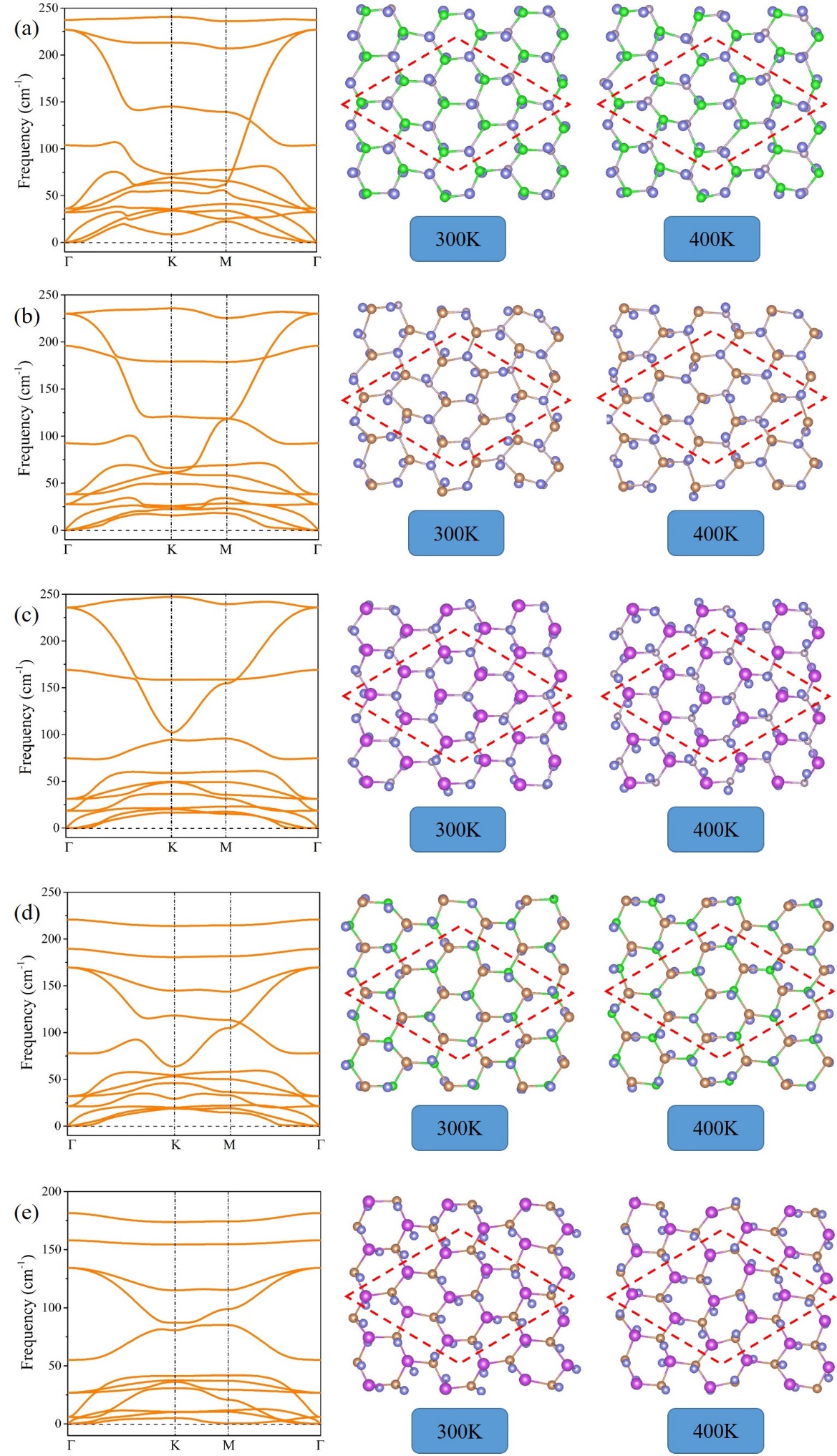


Figure S1. Phonon spectra and molecular dynamics (MD) simulations for a 3×3 supercell at 300K and 400K of ABI_2_ monolayers. (a) PAsI_2_, (b) PSbI_2_ (c) PBiI_2_ (d) AsSbI_2_ and (e) SbBiI_2_ monolayers.


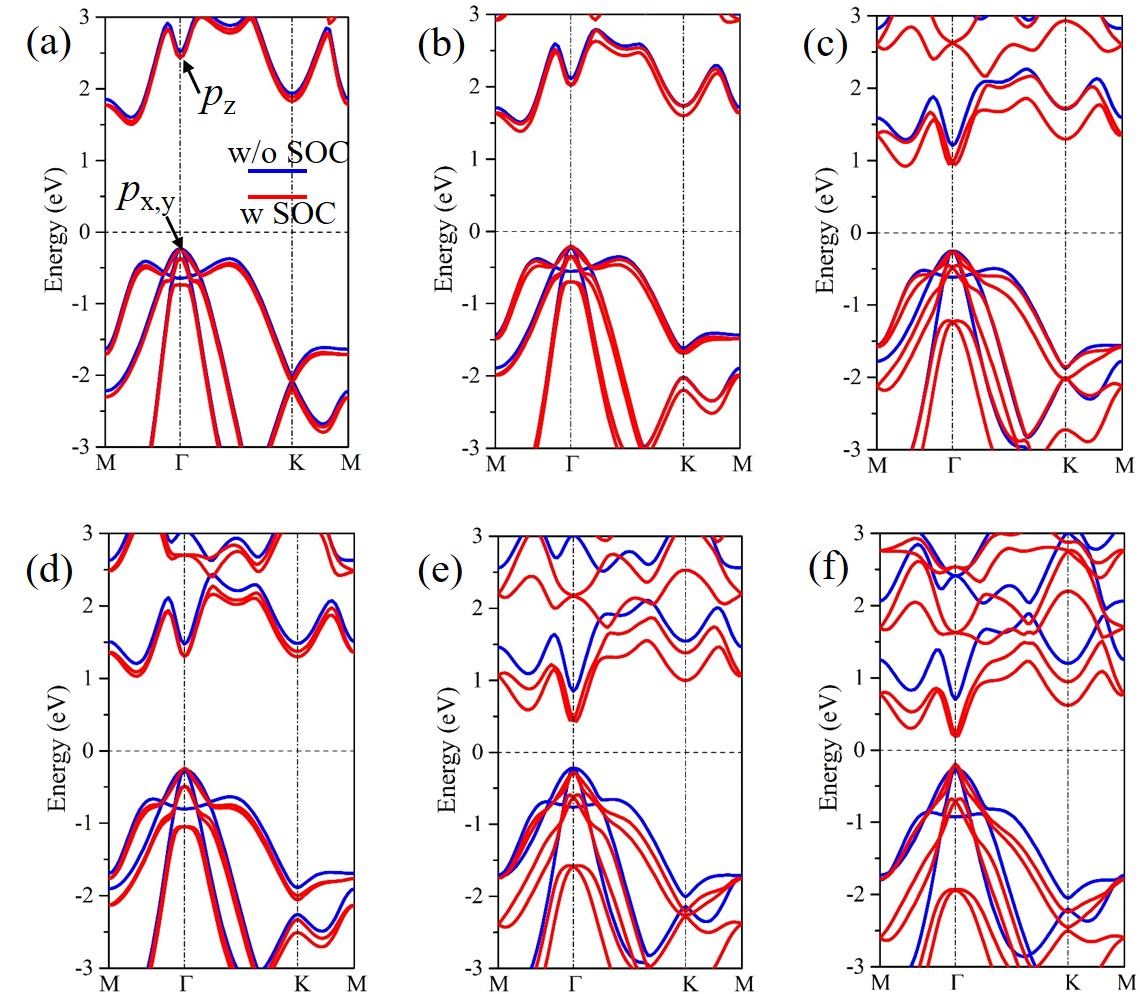


Figure S2. Calculated band structures of (a) PAs, (b) PSb, (c) PBi, (d) AsSb, (e) AsBi and (f) SbBi monolayers. The blue and red line represent the band structure without and with SOC, respectively.


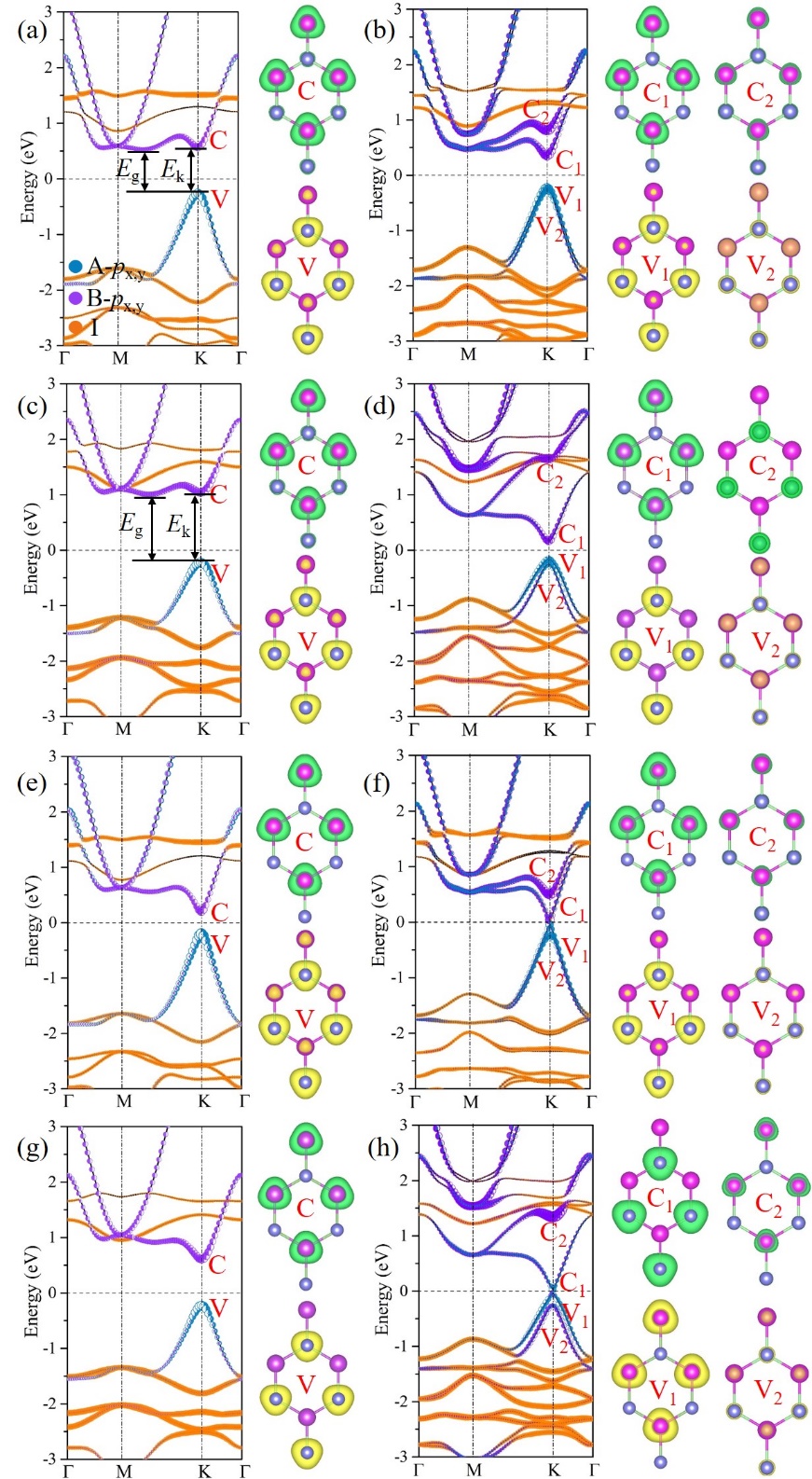


Figure S3. Band structures with orbital projection and partial charge density of VBM and CBM. (a) PSbI_2_ monolayer without SOC, (b) PSbI_2_ monolayer with SOC, (c) PBiI_2_ monolayer without SOC, (d) PBiI_2_ monolayer with SOC, (e) AsSbI_2_ monolayer without SOC, (f) AsSbI_2_ monolayer with SOC, (g) AsBiI_2_ monolayer without SOC, and (h) AsBiI_2_ monolayer with SOC.


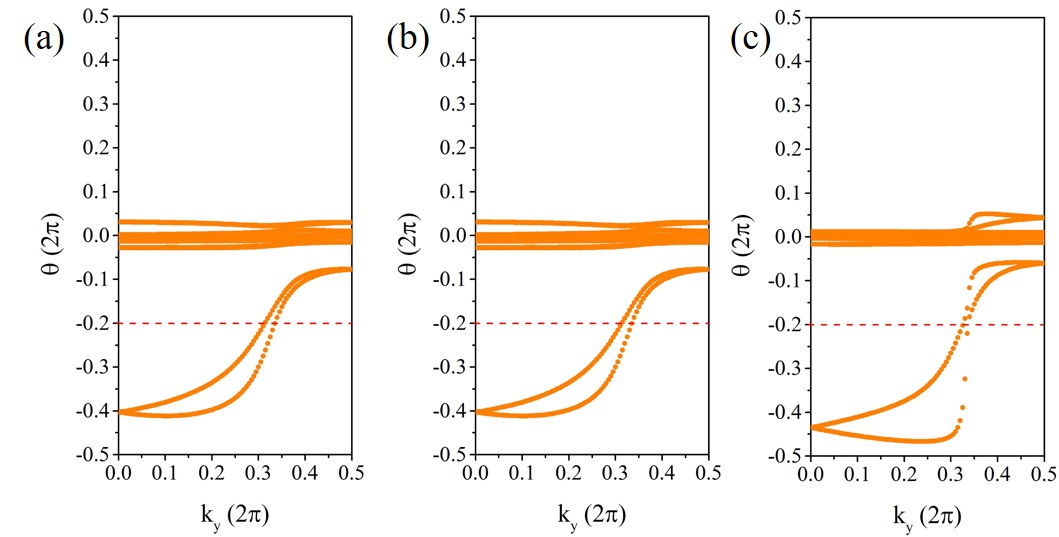


Figure S4. Evolution of the Wannier charge centers (WCCs) along *k*_y_ for (a) PSbI_2_ (b) PBiI_2_ and (c) AsSbI_2_ monolayers.


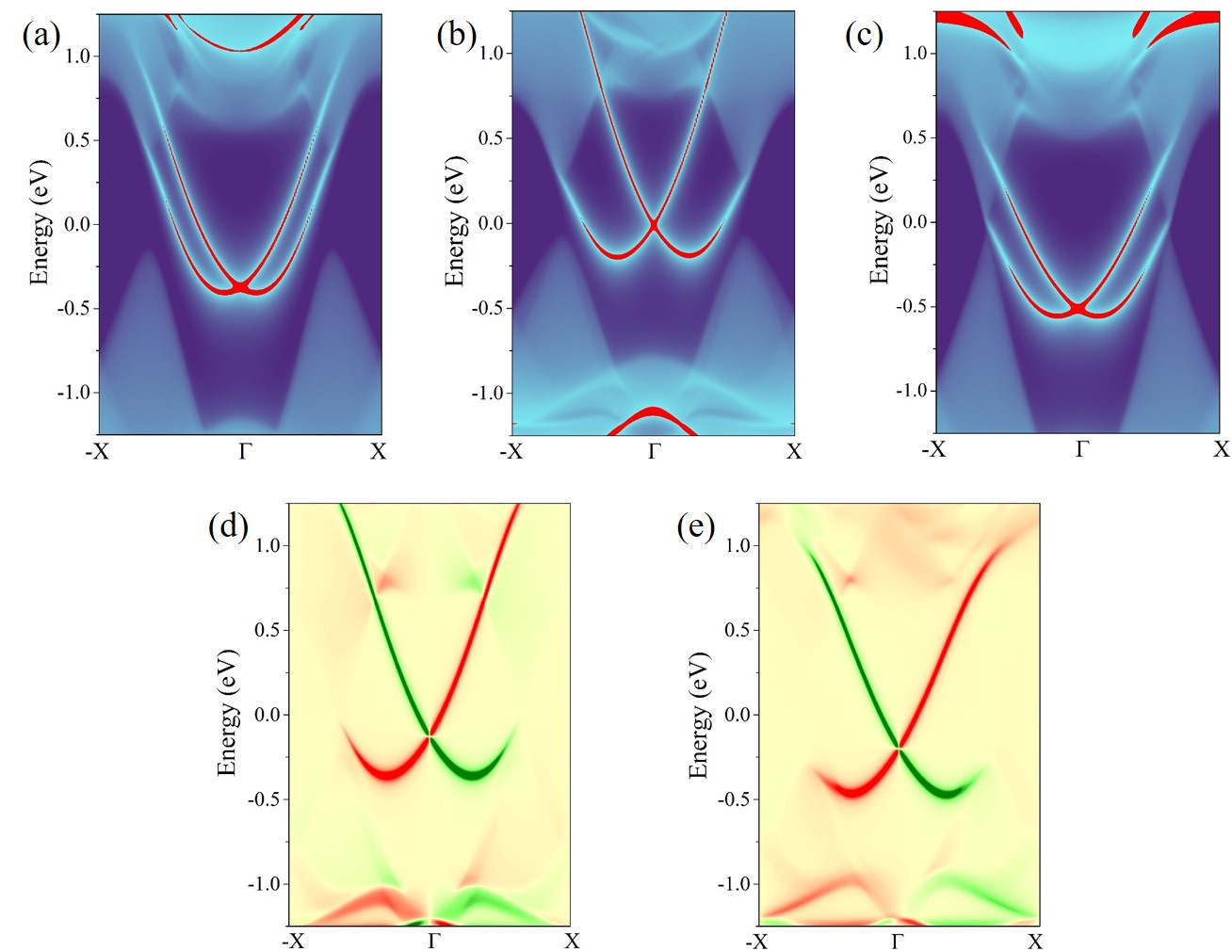


Figure S5. Calculated semi-infinite edge states of (a) PSbI_2_ (b) PBiI_2_ and (f) AsSbI_2_ monolayers. The edge states with spin projection of (d) AsBiI_2_ and (e) SbBiI_2_ monolayers.


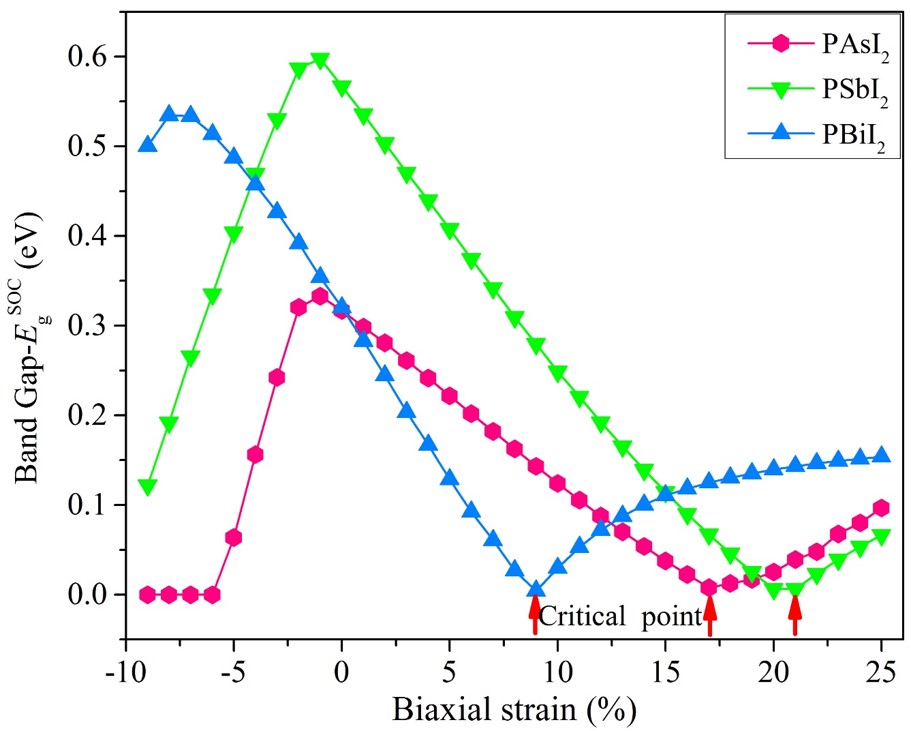


Figure S6. Variation of band gap (*E*_g_^SOC^) as a function of biaxial strain for PAsI_2_, PSbI_2_, and PBiI_2_ monolayers.


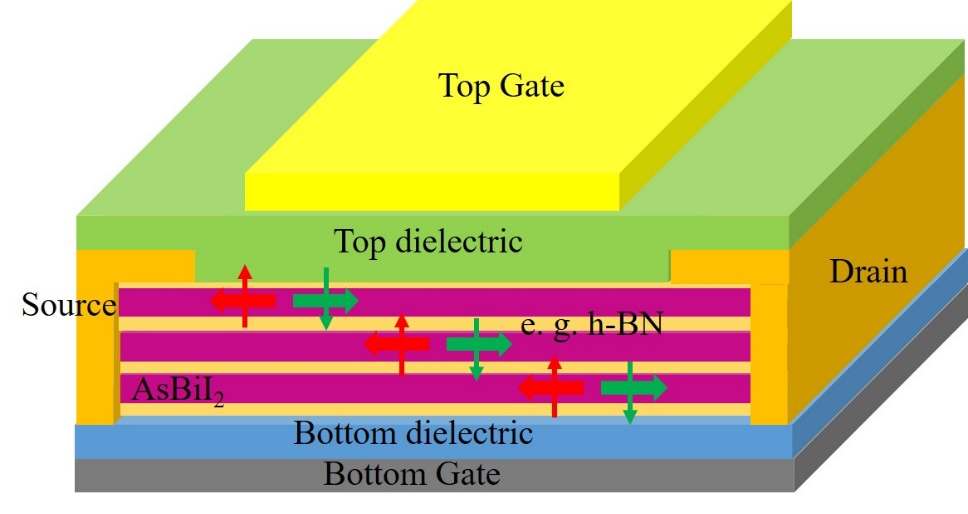


Figure S7. Schematic of vdW-TFET based on AsBiI_2_ monolayer and *h*-BN substrate.


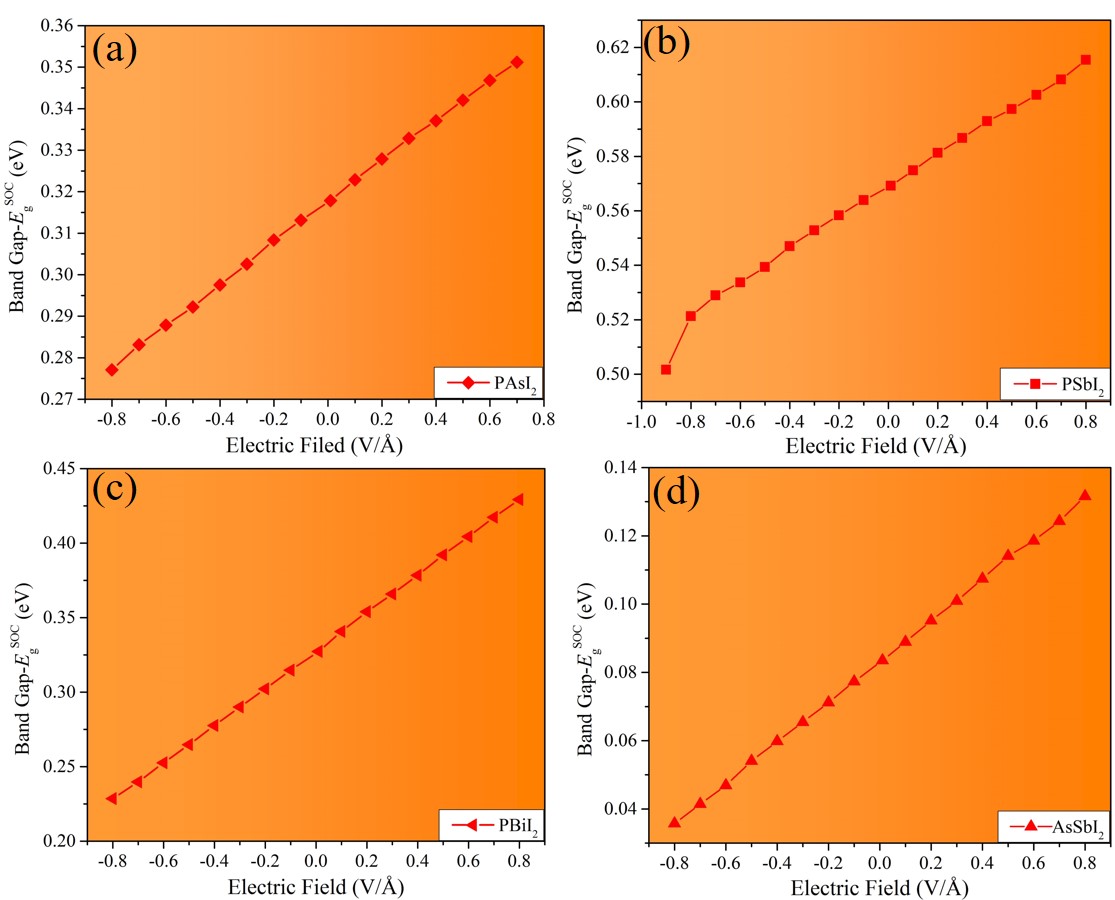


Figure S8. Variation of band gap (*E*_g_^SOC^) as a function of external electric field for (a) PAsI_2_, (b) PSbI_2_, (c) PBiI_2_ and (d) AsSbI_2_ monolayers, respectively.


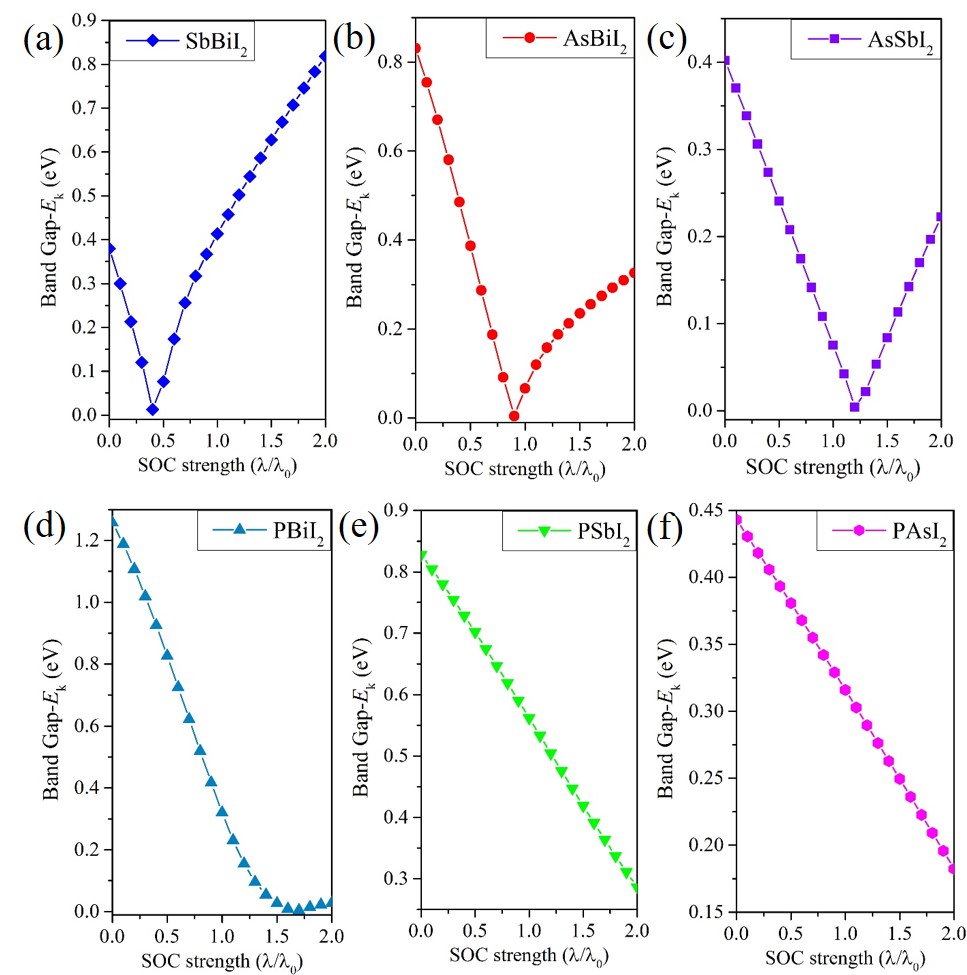


Figure S9. The band gap at the K point of (a) SbBiI_2_, (b) AsBiI_2_, (c) AsSbI_2_, (d) PBiI_2_, (e) PSbI_2_, and (f) PAsI_2_ monolayers as a function of SOC strength from zero to the value set a 2.


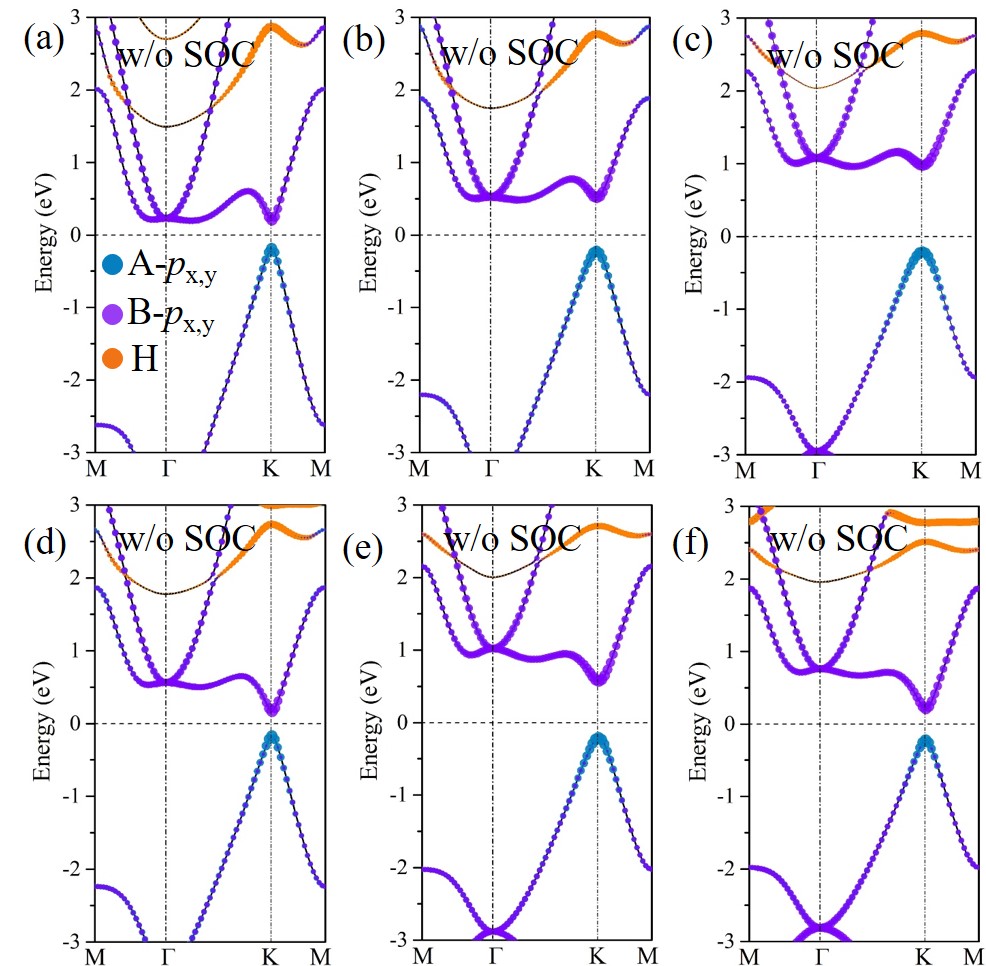


Figure S10. Orbital-resolved band structures of (a) PAsH_2_, (b) PSbH_2_, (c) PBiH_2_, (d) AsSbH_2_, (e) AsBiH_2_ and (f) SbBiH_2_ monolayers without SOC.


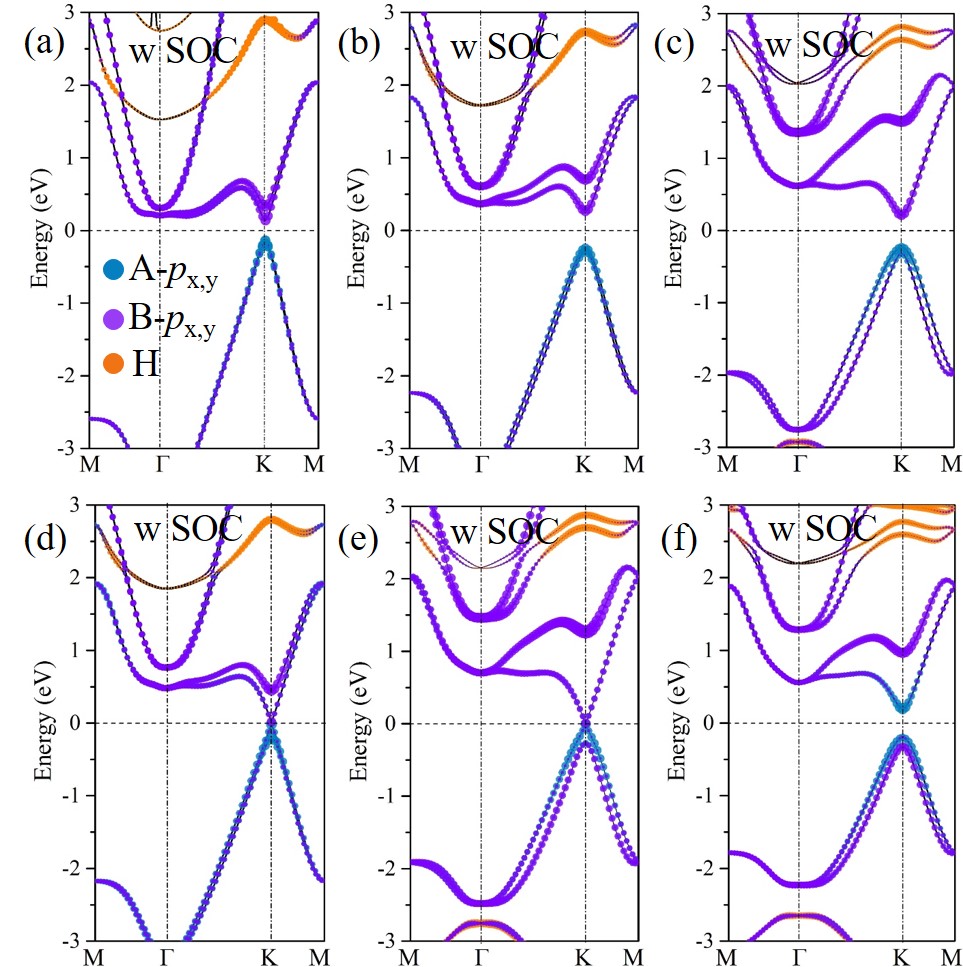


Figure S11. Orbital-resolved band structures of (a) PAsH_2_, (b) PSbH_2_, (c) PBiH_2_, (d) AsSbH_2_, (e) AsBiH_2_ and (f) SbBiH_2_ monolayers with SOC.


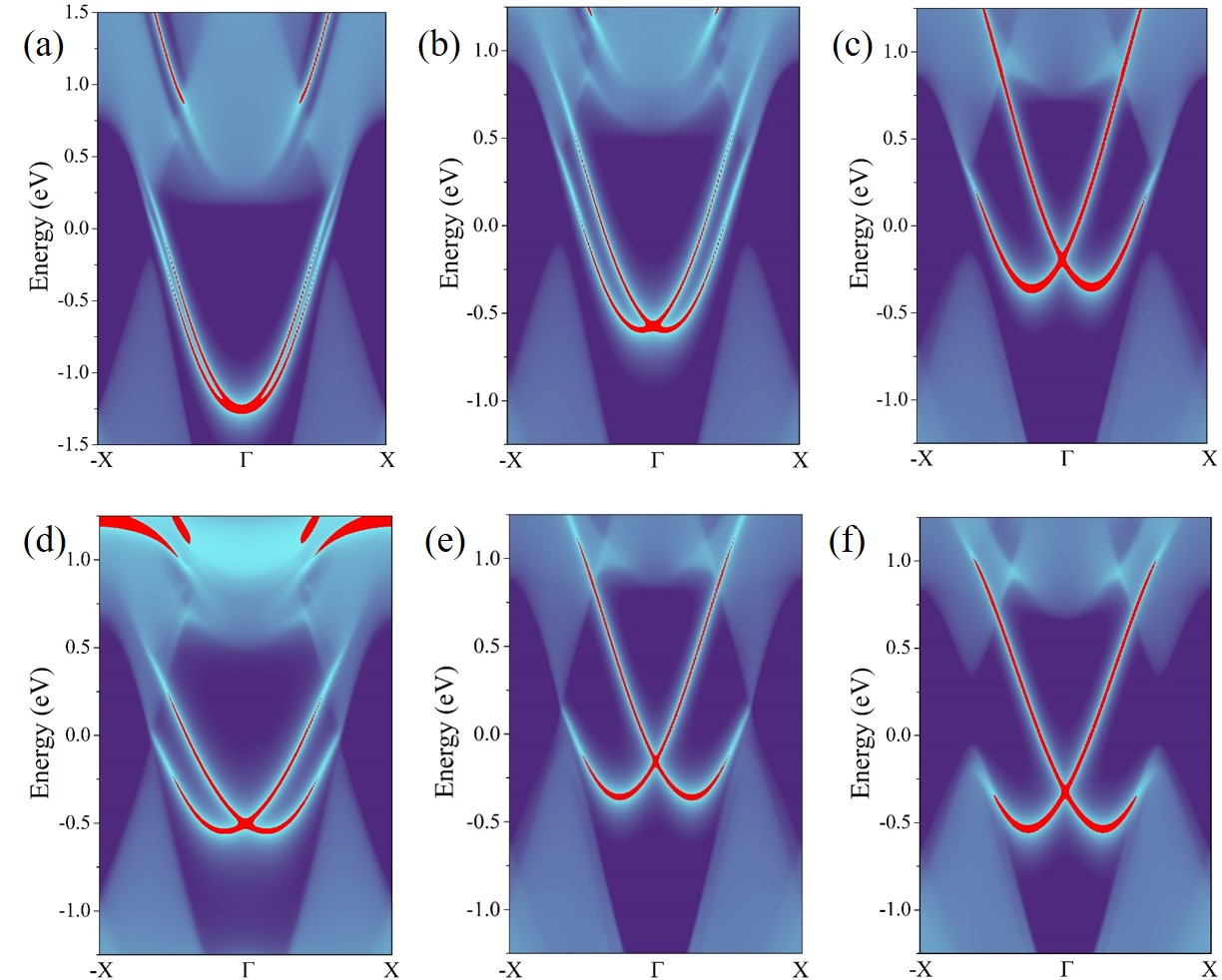


Figure S12. Calculated semi-infinite edge states of (a) PAsH_2_, (b) PSbH_2_, (c) PBiH_2_, (d) AsSbH_2_, (e) AsBiH_2_ and (f) SbBiH_2_ monolayers.


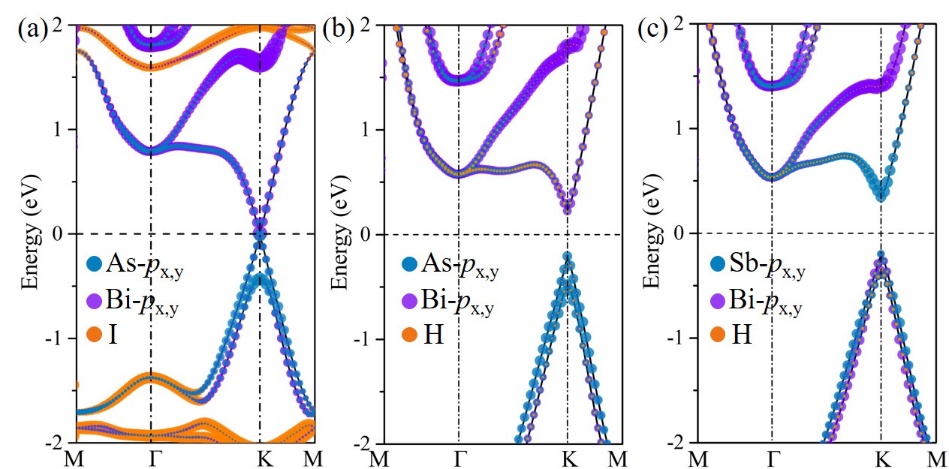


Figure S13. Orbital-resolved band structures of (a) AsBiI_2_, (b) AsBiH_2_, (c) SbBiH_2_ monolayers with SOC, which are calculated based on hybrid HSE06 functional.
